# Increased brain glucose metabolism after anodal left DLPFC–right shoulder tDCS in ASD: an open-label pilot F-18 FDG PET/CT study

**DOI:** 10.1186/s12888-026-08418-x

**Published:** 2026-07-20

**Authors:** Keattichai Keeratitanont, Daris Theerakulpisut, Narong Auvichayapat, Chanyut Suphakunpinyo, Niramol Patjanasoontorn, Somsak Tiamkao, Wiyada Punjaruk, Orathai Tunkamnerdthai, Paradee Auvichayapat

**Affiliations:** 1 Department of Radiology and Nuclear Medicine, Faculty of Medicine, Burapha University, Chonburi, 20131 Thailand; 2 Division of Nuclear Medicine, Department of Radiology, Faculty of Medicine, Khon Kaen University, Khon Kaen, 40002 Thailand; 3 Division of Pediatric Neurology, Department of Pediatrics, Faculty of Medicine, Khon Kaen University, Khon Kaen, 40002 Thailand; 4 Division of Child Development, Department of Pediatrics, Faculty of Medicine, Khon Kaen University, Khon Kaen, 40002 Thailand; 5 Division of Child and Adolescent Psychiatry, Department of Psychiatry, Faculty of Medicine, Khon Kaen University, Khon Kaen, 40002 Thailand; 6 Division of Neurology, Department of Medicine, Faculty of Medicine, Khon Kaen University, Khon Kaen, 40002 Thailand; 7 Department of Physiology, Faculty of Medicine, Khon Kaen University, Khon Kaen, 40002 Thailand; 8 Noninvasive Brain Stimulation Research Group of Thailand, Faculty of Medicine, Khon Kaen University, Khon Kaen, 40002 Thailand

**Keywords:** Brain glucose metabolism, Positron Emission Computed Tomography (PET/CT), Autism Spectrum Disorder (ASD), Transcranial Direct Current Stimulation (tDCS), Dorsolateral Prefrontal Cortex (DLPFC)

## Abstract

**Background:**

Anodal transcranial direct current stimulation (tDCS) using an extracephalic montage has demonstrated clinical efficacy in improving social functioning in individuals with autism spectrum disorder (ASD), however its neurobiological mechanisms remain poorly understood.

**Methods:**

**I**n this open-label pilot study, six adolescents with high-functioning ASD underwent five consecutive daily sessions of 2-mA anodal tDCS, left dorsolateral prefrontal cortex (DLPFC)–right shoulder montage. Therapeutic effects were subsequently assessed by comparing pre- and post-intervention brain glucose metabolism via F-18 FDG PET/CT in 26 regions of interest and clinical symptoms via the Autism Treatment Evaluation Checklist (ATEC) social subscale.

**Results:**

Following the intervention, metabolic increases exceeding the predefined 10% threshold were observed in four key brain regions: prefrontal lateral, parietal inferior, temporal mesial, and cerebellum (FDR-adjusted *p* < 0.05). Furthermore, negative correlations were observed between ATEC social scores and metabolic increases in the left prefrontal lateral (*r* = − 0.832, *p* = 0.040) and cerebellum (*r* = − 0.868, *p* = 0.025). No adverse events were reported.

**Conclusion:**

The metabolic increases were observed after the intervention in key regions, particularly the prefrontal lateral and cerebellum, which correlated with reduced social impairment in adolescents with ASD. These preliminary, exploratory findings warrant evaluation in larger randomized sham-controlled trials.

**Clinical trial registration:**

ClinicalTrials.gov Registration (TCTR) on 5 July 2021 (ID: TCTR20210705005; https://trialsearch.who.int/Trial2.aspx?TrialID=TCTR20210705005).

**Supplementary Information:**

The online version contains supplementary material available at https://doi.org/10.1186/s12888-026-08418-x.

## Introduction

Autism Spectrum Disorder (ASD) is increasingly conceptualized not merely as a localized neural dysfunction, but as a pervasive synaptopathy, characterized by aberrant long-range functional connectivity and disrupted neural synchronization [1]. Despite the rising global prevalence of ASD, current pharmacological interventions remain largely supportive rather than curative, failing to address these underlying neurophysiological deficits [2]. Consequently, there is an urgent imperative for novel therapeutic strategies capable of directly modulating these dysregulated brain networks.

Transcranial Direct Current Stimulation (tDCS) has emerged as a promising non-invasive neuromodulatory technique for addressing these deficits. Recent meta-analyses corroborate that tDCS significantly ameliorates core ASD symptoms, particularly improving social functioning as indexed by the ATEC social subscale [3–6]. Crucially, the therapeutic efficacy of tDCS is highly montage-dependent. While conventional bicephalic montages are predominantly utilized, recent meta-analytic data evaluating clinical outcomes indicate that anodal stimulation of the left dorsolateral prefrontal cortex (DLPFC) paired with an extracephalic reference has been associated with more favorable clinical outcomes [7]. Specifically, the Left DLPFC–Right Shoulder montage (Anode: F3, Cathode: Right Shoulder) has garnered substantial clinical interest due to its reported clinical benefits across multiple studies [8–12]. Although the precise biophysical mechanisms underpinning the superiority of the extracephalic montage remain to be fully elucidated, computational modeling provides a compelling rationale. These models suggest that placing the return electrode extra cephalically mitigates scalp shunting and generates a unique, vertical whole-head electric field. This configuration is projected to drive current density through deeper cortical and subcortical structures, as well as brainstem pathways, along the trajectory toward the extracephalic reference [13–15].Consequently, regions along this trajectory (such as the mesial temporal lobe and cerebellum) are more likely to be neuromodulated.

Despite the well-documented clinical benefits, the precise neurobiological mechanisms driving these improvements remain to be fully elucidated. Based on the existing evidence, we hypothesize that electrical stimulation from this montage modulates the dysregulated brain networks underlying sociability in ASD, although the specific neural targets involved have yet to be definitively identified.

Previous magnetic resonance spectroscopy (MRS) studies have identified neurochemical alterations, such as increased glial-related metabolites (e.g., myo-inositol) in the DLPFC and locus coeruleus, following this montage stimulation [11]. While informative, these Region-of-Interest (ROI) based findings are limited in their ability to capture network-wide neuromodulatory effects, which are central to the pathophysiology of ASD [16, 17].

To bridge this knowledge gap, F-18 fluorodeoxyglucose positron emission tomography/computed tomography (FDG PET/CT) offers a distinct advantage by enabling whole-brain assessment of cerebral glucose metabolism. As glucose metabolism serves as a reliable proxy for synaptic activity and neuronal energetics, it closely aligns with functional connectivity measures [18, 19]. Thus, examining metabolic topography offers a novel pathway to visualize how the Left DLPFC–Right Shoulder montage may be associated with changes in functional brain networks on a global scale.

Given the limited understanding of the metabolic underpinnings of this extracephalic montage, this preliminary, open-label pilot study investigates cerebral glucose metabolism changes in adolescents with ASD utilizing F-18 FDG PET/CT, we aimed to explore regional metabolic alterations—specifically focusing on regions with > 10% increased uptake—and to examine their potential association with clinical improvements in the ATEC social subscale. This work serves as a proof-of-concept investigation to identify candidate neural substrates and generate hypotheses for future randomized controlled trials.

## Materials and methods

### Study design

This study employed a before-and-after design with six participants diagnosed with ASD. The one-month protocol comprised three distinct phases:

#### Baseline

Comprehensive evaluation including regional brain glucose metabolism via PET/CT and Autism Treatment Evaluation Checklist (ATEC) scoring.

#### Intervention

Five consecutive days of transcranial direct current stimulation (tDCS).

#### Post-intervention

**Fig. 1:**
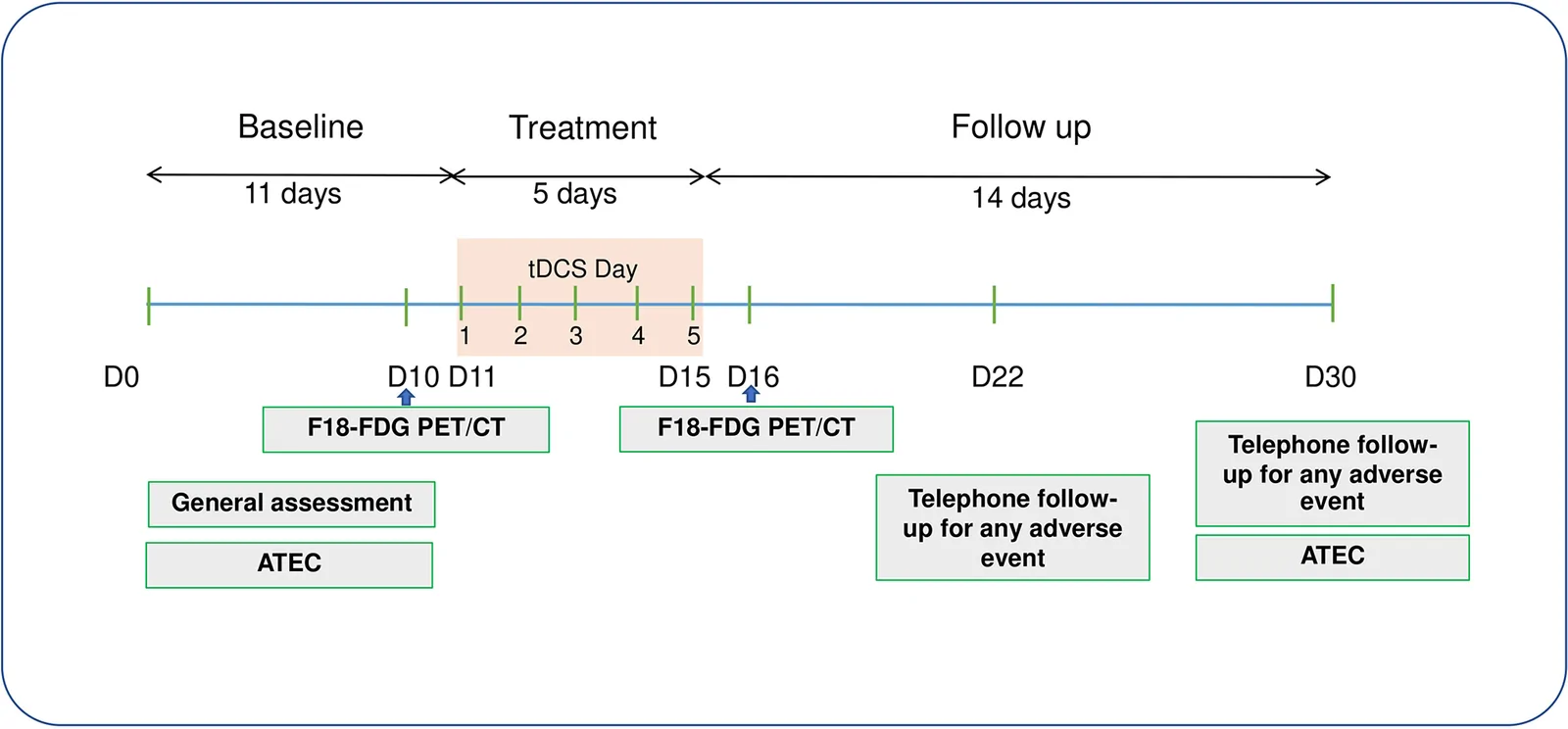
Timeline of the study

Brain glucose metabolism reassessment one day after treatment, followed by ATEC re-evaluation at 14 days (Fig. 1).

The timing of post-intervention assessments was strategically determined based on the distinct physiological natures of neurometabolic changes and behavioral adaptations. The FDG-PET/CT scan was performed one day post-intervention to capture immediate, acute alterations in brain glucose metabolism prior to any potential washout effects, aligned with established post-tDCS neuroimaging timelines [20]. Conversely, the final behavioral assessment using the Autism Treatment Evaluation Checklist (ATEC) was scheduled at Day 14 post-intervention. This 14-day window was selected because clinically meaningful behavioral improvements observed following tDCS in individuals with ASD typically accumulate gradually, starting around Day 7 and reaching a stable plateau around two weeks post-stimulation [8–11]. Additionally, the ATEC is designed to measure ‘current typical behavior’ over a 2-to-4-week observational window; evaluating it immediately on Day 1 would capture transient day-to-day behavioral fluctuations rather than sustained post-intervention behavioral changes. Furthermore, this timeline maintains methodological consistency with prior standardized protocols conducted by our research group, ensuring cross-study comparability.

To maintain ecological validity, participants continued their usual daily routines without additional cognitive or behavioral interventions. Caregivers documented daily activities to ensure no atypical events influenced the outcomes.

### Participant recruitment and informed consent

Participants were recruited and enrolled between July 7, 2021, and July 23, 2021 via advertisements in Khon Kaen, Thailand. ASD diagnosis was made by a child psychiatrist (N.P.) according to DSM-5 criteria using the Autism Diagnostic Observation Schedule-2 (ADOS-2) [21]. High-functioning ASD was defined as ASD without co-occurring intellectual impairment. This was confirmed by a Nonverbal IQ score of ≥ 70 on the Test of Nonverbal Intelligence, Fourth Edition (TONI-IV), together with preserved functional abilities, including effective communication, performance of activities of daily living, and independent living skills, according to the criteria described by Ratsapbhayakul et al. [12]. Inclusion criteria included: (1) age 15–24 years; (2) normal vision and hearing; and (3) informed consent from both participant and caregiver. Exclusion criteria included: (1) Medical/Safety: Skull defects, metallic implants, skin lesions at electrode sites, pregnancy, or claustrophobia. (2) Neurological: Severe disorders (e.g., tumors, infections), uncontrollable epilepsy, or inability to remain still during scanning. (3) Confounding Factors: Use of neuropsychiatric medications, substances (alcohol, caffeine, cannabis), or alternative therapies (HBOT, craniosacral therapy); sleep deprivation; or neuromodulation within the past year. (4) Study Integrity: Receipt of new concurrent treatments/training or uncooperative caregivers.

Screening procedure:

Initial screening included a medical history and physical examination by a neurologist (S.T. or N.A.) to identify comorbidities. Eligible participants then underwent structural MRI and a formal functional assessment by a child psychiatrist (N.P.).

#### Transcranial direct current stimulation

tDCS was administered using a battery-powered Soterix Medical stimulator (Model 1300 A, NY, USA). Participants received 20-minute daily sessions of 2-mA current for five consecutive days, with 30-second ramp-up and ramp-down periods. Following the international 10–20 system, the anode was placed over the left DLPFC (F3) using 5 × 7 cm saline-soaked sponge electrodes, while the cathode was positioned on the right shoulder. This protocol aligns with previously established parameters for ASD [8–12].

### Measures

#### FDG-PET/CT procedure and analysis

##### Image acquisition

Participants fasted for ≥ 6 h before receiving an intravenous injection of approximately 7 mCi F-18 FDG (95% purity). Following a 45-minute uptake period in a quiet, dimly lit room, scans were performed using a Discovery 690 PET/CT scanner (GE Healthcare, USA). A low-dose CT was acquired for attenuation correction (120 kV, 10 mA), followed by a 3D PET emission scan. Images were reconstructed using an ordered-subset expectation maximization (OSEM) algorithm.

##### Image processing and ROI definition

Data were processed on a GE Xeleris workstation (v4.0). Automated segmentation defined 26 cortical regions of interest (ROIs), including bilateral prefrontal lateral, prefrontal medial, sensorimotor, anterior cingulate, posterior cingulate, precuneus, parietal superior, parietal inferior, occipital lateral, primary visual, temporal lateral, temporal mesial, as well as the whole cerebellum and pons.

##### Quantification strategy

To minimize inter-individual physiological variability and avoid the limitations of absolute standardized uptake values (SUVs) in adolescents [22, 23], a semi-quantitative regional ratio approach was utilized. The pons was selected as the reference region—rather than the cerebellum or whole brain—to avoid potential ASD-related metabolic confounding [24, 25].

##### Outcome measures and study timeline

Metabolic activity was expressed as the ratio of mean ROI uptake to mean pons uptake. Scans were obtained at two time points: baseline (one day pre-tDCS) and post-intervention (one day after the fifth tDCS session). To minimize potential bias in imaging outcome assessment, quantitative PET analysis was conducted using Q.Brain software (GE Healthcare), an automated, operator-independent platform for image segmentation and quantification. ROI extraction and semi-quantitative analyses were fully automated, thereby reducing observer-dependent variability and ensuring that image processing remained effectively blinded to participants’ clinical outcomes. Furthermore, the nuclear medicine physician responsible for quantitative analysis (D.T.) was not involved in tDCS administration and remained unaware of individual clinical outcomes throughout the analytical process, thereby further mitigating assessment-related bias.

##### Predefined objective threshold

Percentage change in brain glucose metabolism ratio was calculated as:

$$\begin{aligned}&\:\mathrm{P}\mathrm{e}\mathrm{r}\mathrm{c}\mathrm{e}\mathrm{n}\mathrm{t}\mathrm{a}\mathrm{g}\mathrm{e}\:\mathrm{c}\mathrm{h}\mathrm{a}\mathrm{n}\mathrm{g}\mathrm{e}\:= \cr&\quad\left(\frac{{BGM}_{posttDCS}-{BGM}_{pretDCS}}{{BGM}_{pretDCS}}\right)\times\:100\end{aligned}$$

In the absence of established metabolic change thresholds for neuromodulation in ASD, we intentionally adopted a conservative threshold of > 10% change to isolate regions of interest (ROIs). While prior psychiatric neuroimaging studies have utilized lower thresholds (ranging from 1 to 9%) [26], we elected to apply the more stringent > 10% threshold derived from established test-retest reliability data [27]. Given our restricted sample size, this conservative methodological approach was implemented to minimize Type I errors and ensure that the identified regional changes represented substantial metabolic shifts rather than normal physiological variance or scanner noise. To control for the inflation of Type I error rates resulting from multiple comparisons, the Benjamini–Hochberg FDR procedure was applied to the p-values of ROIs that met or exceeded this 10% threshold.

#### Autism treatment evaluation checklist (ATEC)

The validated Thai version of the ATEC was used to evaluate clinical symptom changes across four domains: (1) Speech/Language/Communication, (2) Sociability, (3) Sensory/Cognitive Awareness, and (4) Health/Physical/Behavioral. Total scores range from 0 to 179, with higher scores indicating greater severity. The Thai-ATEC exhibits excellent reliability (ICC = 0.97) and diagnostic accuracy (AUC 0.85–0.90), with established cut-off scores for mild (≤ 38) and severe (≥ 68) symptoms [28]. For each participant, two caregivers completed the checklist at baseline and 14 days post-intervention, resulting in an approximately 30-day interval between clinical assessments.

Prior to study initiation, a structured rater calibration session was conducted using standardized behavioral vignettes to operationally define each scoring level, thereby enhancing inter-rater reliability and minimizing systematic scoring variability. Caregivers were explicitly instructed to report outcomes with maximal accuracy and objectivity, irrespective of perceived treatment effects, in order to mitigate expectancy and social desirability biases. To further reduce individual subjective bias, composite scores were calculated as the mean of independent ratings obtained from two caregivers.

Outcome assessments were performed on Day 14 post-treatment to limit retrospective recall bias. To support contemporaneous and evidence-based reporting, caregivers were required to maintain daily behavioral logs, which served as an objective reference during questionnaire completion and reduced reliance on global impressions. Furthermore, to ensure standardized interpretation of questionnaire items, a child development physician with expertise in ATEC assessment (CS) provided neutral, item-by-item clarification to all caregivers, thereby minimizing information bias and enhancing measurement validity.

#### Adverse events

Caregivers were instructed to report any adverse events, as well as any other signs and symptoms, on a daily basis following treatment. Concurrently, participants were under close observation by physicians during each stimulation session. This self-reporting process was concluded 7 and 14 days subsequent to the final stimulation session.

### Statistical analysis

Sample size was determined using data from Buchsbaum et al. (2001), where a mean difference of 0.09 (SD = 0.071) in relative brain glucose metabolism ratio was reported. To achieve 90% power (alpha = 0.05), six participants were required. Although 10 were recruited to account for a 40% dropout rate, six completed the full protocol and were included in the final per-protocol analysis. Statistical analyses were performed using IBM SPSS Statistics, version 26.0 (IBM Corp., Armonk, NY, USA). Due to the small sample size (*n* = 6), non-parametric tests were employed. Descriptive statistics are reported as mean ± standard deviation (SD).

For the neuroimaging data, an exploratory ROI-based analysis was performed. To descriptively isolate regions with clinically meaningful shifts, ROIs demonstrating a metabolic change greater than 10% were first identified. Subsequently, False Discovery Rate (FDR) correction was applied strictly within this subset of ROIs to control for multiple comparisons. We explicitly note that this two-step sequence introduces a potential selection bias (circular analysis), as the statistical testing was conditional on the initial magnitude filtering. Therefore, the corrected p-values presented herein must be interpreted as preliminary, exploratory, and hypothesis-generating rather than definitive.

Effect sizes for Wilcoxon signed-rank tests were calculated using r = Z / √N and interpreted as small (0.1), medium (0.3), or large (0.5). Exploratory associations between metabolic changes and ATEC subscale scores were assessed using Spearman’s rank correlation (*p*). Given the pilot nature of the study, p> |0.7| over p-values, with scatter plots used to ensure results were not outlier-driven.

## Results

### Participant characteristics

**Fig. 2:**
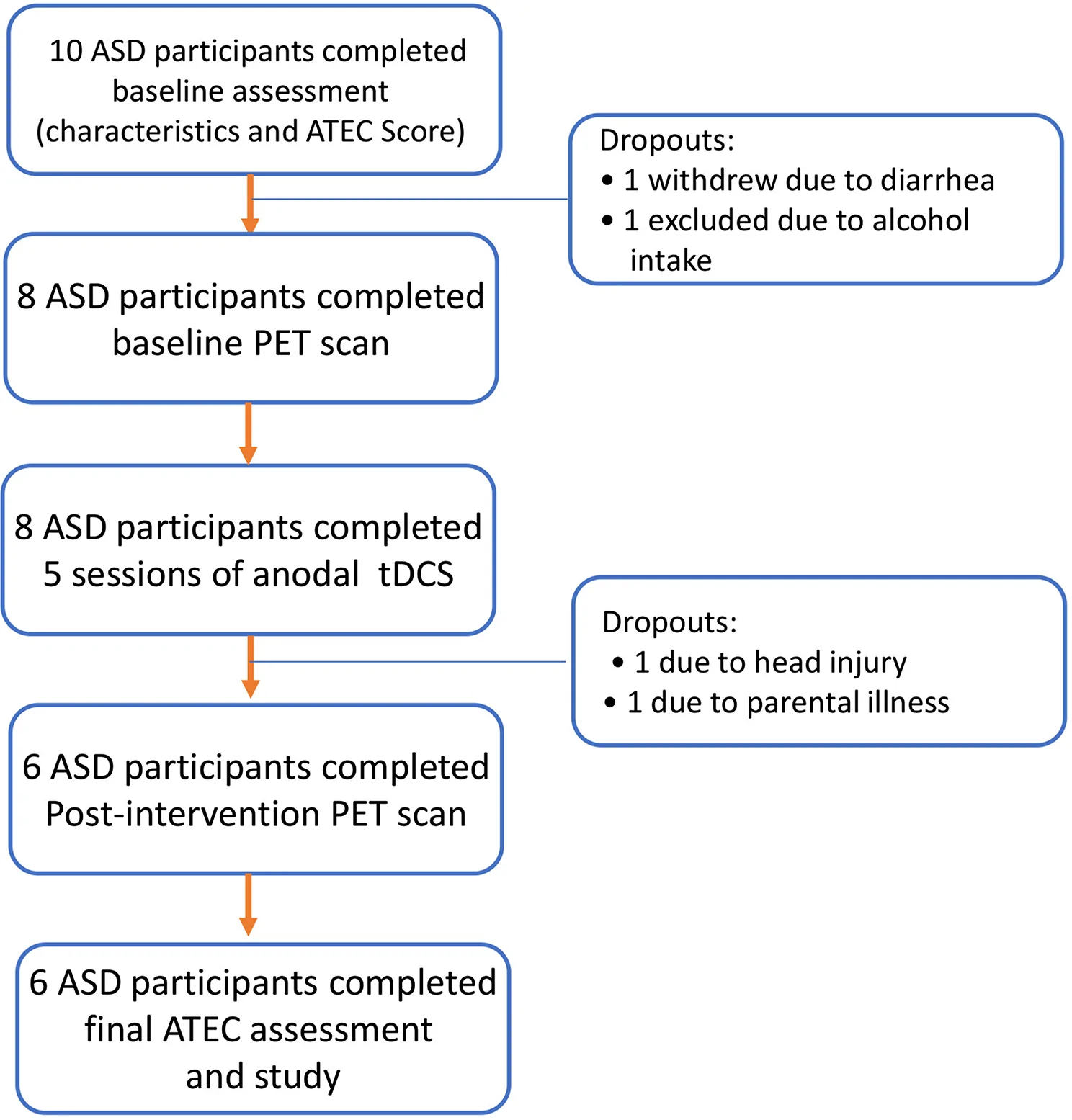
Flow of participants

This study (TCTR20210705005) investigated the effects of tDCS in individuals with ASD. A total of 10 individuals with ASD were enrolled and completed baseline assessments, including demographic characteristics and ATEC scoring. Two participants did not proceed to imaging: one withdrew due to gastrointestinal illness, and one was excluded because of alcohol intake, leaving eight participants for baseline PET imaging. All 8 participants subsequently completed five sessions of anodal tDCS. During the intervention period, two additional participants discontinued: one following a head injury and another due to parental illness. Consequently, six participants completed post-intervention PET imaging and the final ATEC assessment, which constituted the final study sample (Fig. 2).

The final cohort (*n* = 6) comprised five males (83%) and one female, aged 15 to 22 years (mean: 17.8; median: 17.5). The majority were left-handed (66.7%). Clinical history indicated a mean diagnosis age of 4.2 years (range: 1.5–11) with primarily school-based behavioral interventions. Birth histories showed a 50% cesarean section rate and largely uncomplicated pregnancies (one case of pre-eclampsia), with mean maternal and paternal ages of 32 and 38 years, respectively (Table 1).

**Table 1:** Demographic data of participants (*N* = 6)

ID	ASD01	ASD02	ASD03	ASD05	ASD06	ASD07
Sex	F	M	M	M	M	M
Age (years)	19	15	16	18	22	17
TONI-IV index score	100	84	87	74	82	86
Handedness	R	L	R	L	L	L
Age of diagnosis	3.5	11	3.25	4.25	1.92	1.5
Parturition	C/S	C/S	N/L	NL	N/L	C/S
Maternal age at parturition	33	28	34	26	30	40
Paternal age at parturition	40	38	34	30	35	50
History of pregnancy	Normal	PE	Normal	Normal	Normal	Normal
Family history for ASD	Yes	No	No	No	No	No
Behavioral therapy	SB	SB	SB	SB	OT	OT

C/S = cesarean section; F=female; L = left; M = male; N/L=normal labor; OT= occupational therapy; PE = pre-eclampsia; R=right; SB = school base; ASD = autism spectrum disorders; TONI-IV index score = The index score of Test of Nonverbal Intelligence, fourth edition; <70 = very poor, 70–79 = poor, 80–89 = below average, 90–110 = average, 111–120 = above average. Higher score indicates good general intelligence

### Effect of tDCS on brain glucose metabolism

**Fig. 3:**
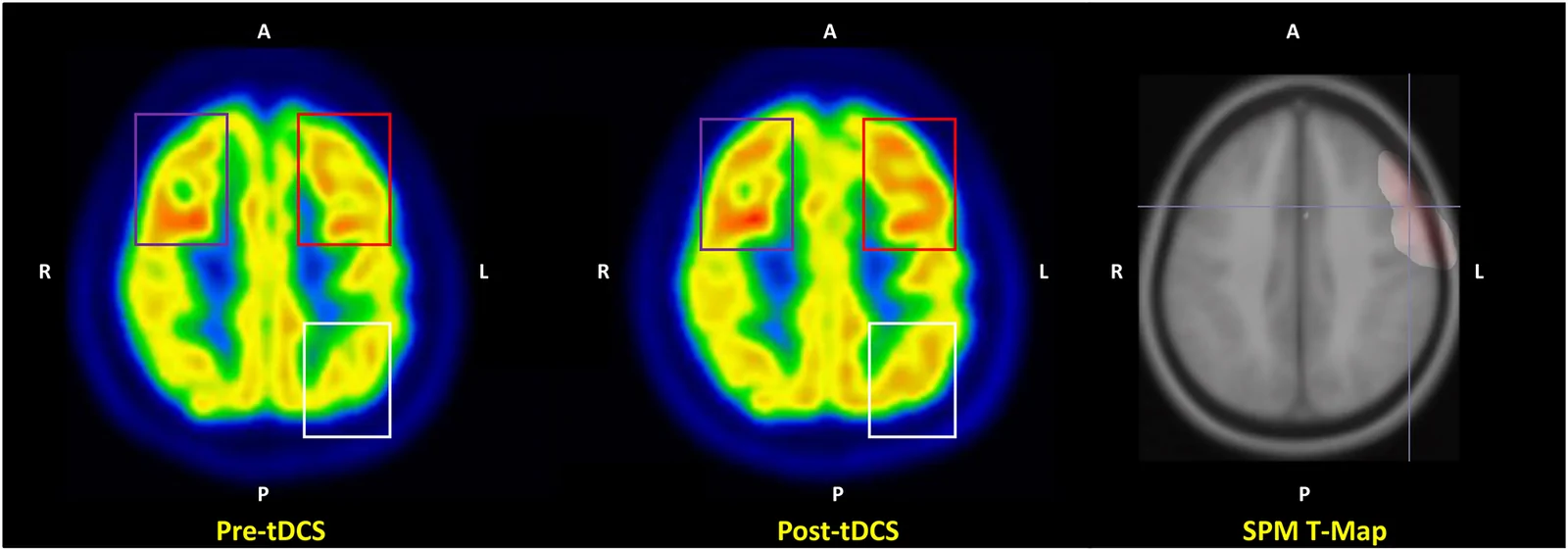
Axial F-18 FDG PET/CT images show increased glucose metabolism predominantly in the left lateral prefrontal (outlined in red) and left parietal regions (outlined in white), with a milder elevation in the right lateral prefrontal region (outlined in purple) following anodal tDCS using a left dorsolateral prefrontal cortex–right shoulder montage. The SPM T-map also shows a significant increase in FDG uptake relative to baseline in the left DLPFC (x = − 45, y = 20, z = 40)

**Fig. 4:**
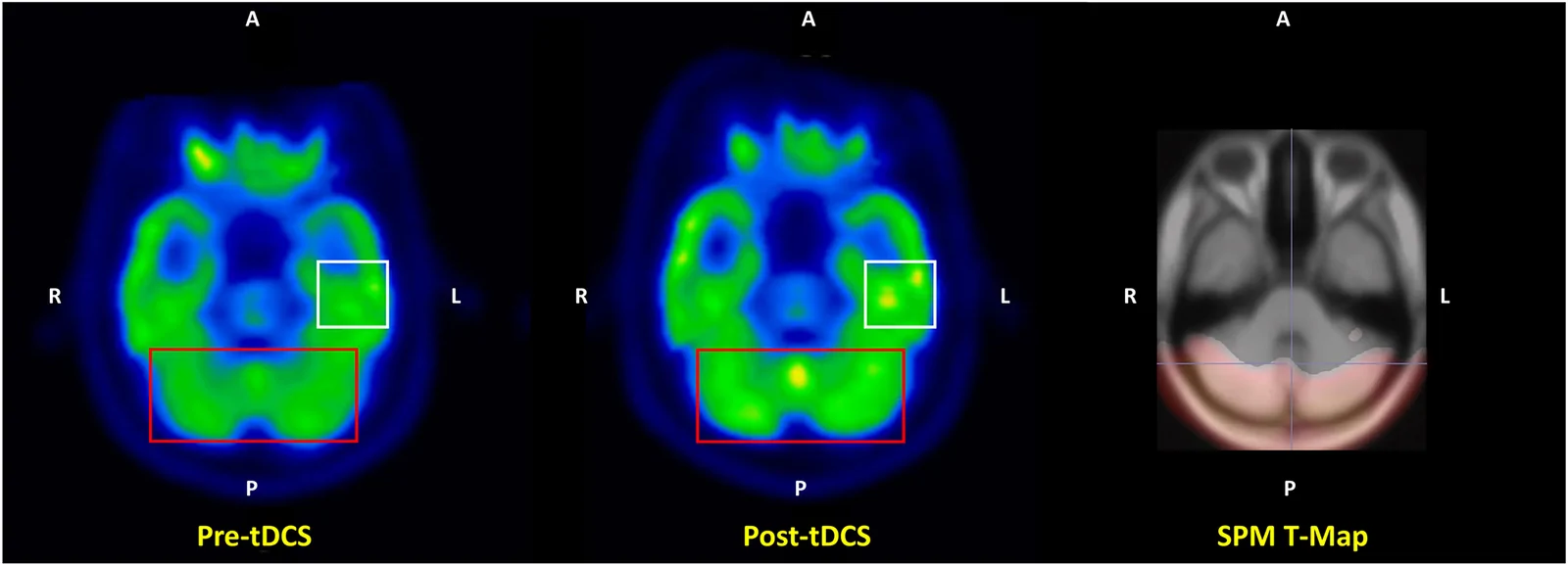
Axial F-18 FDG PET/CT images show increased glucose metabolism in the left mesial temporal region (outlined in white) and the cerebellum (outlined in red) following anodal tDCS using a left dorsolateral prefrontal cortex–right shoulder montage. The SPM T-map also shows a significant increase in FDG uptake relative to baseline in the cerebellum (x = 0, y = -70, z = -45)

Visual assessment suggested metabolic increases in the left frontal, parietal, temporal mesial, and cerebellar regions (Figs. 3 and 4). Quantitative analysis revealed that four ROIs exceeded the predefined 10% threshold left prefrontal lateral (11.61% ± 1.62), left parietal inferior (10.87% ± 1.21), left temporal mesial (10.33% ± 0.86), and the cerebellum (10.12% ± 1.77). The percentage changes in brain glucose metabolism ratios across 26 ROIs are shown in Table 2. Wilcoxon signed-rank tests revealed significant increases across all four regions (Z = -2.201, unadjusted *p* < 0.03). Significance was maintained following Benjamini–Hochberg FDR correction (adjusted *p* = 0.028; effect size = 0.64; Table 3).

**Table 2:** Percentage changes in brain glucose metabolism ratios across regions of interest (ROIs) derived from Q.Brain software in the ASD (*N* = 6)

Region of interest (ROI)	Right Hemisphere								Left Hemisphere							
	ASD01	ASD02	ASD03	ASD05	ASD06	ASD07	Mean	SD	ASD01	ASD02	ASD03	ASD05	ASD06	ASD07	Mean	SD
Prefrontal Lateral	3.33	5.32	2.55	3.55	1.83	4.44	3.50	1.15	10.39	10.47	11.22	15.12	11.56	10.87	11.61*	1.62
Prefrontal Medial	4.20	2.67	9.39	0.60	0.64	4.37	3.65	2.98	7.64	7.26	9.34	5.13	4.91	1.72	6.00	2.44
Sensorimotor	-1.14	-1.97	1.60	-3.37	-1.02	3.57	-0.39	2.31	2.31	2.00	4.23	3.35	3.48	4.57	3.32	0.93
Anterior cingulate	0.76	-1.25	-1.75	-1.32	0.68	-3.51	-1.06	1.47	0.77	0.61	0.57	3.90	8.97	0.58	2.57	3.10
Posterior cingulate	-0.59	0.96	1.60	1.09	-0.54	0.52	0.51	0.82	0.59	3.00	1.59	3.33	0.52	0.51	1.59	1.18
Precuneus	-2.70	-0.57	-1.09	1.90	0.63	-1.75	-0.60	1.51	1.32	1.70	1.09	5.52	2.48	1.66	2.30	1.51
Parietal superior	7.28	1.63	4.35	1.27	3.51	0.58	3.10	2.28	8.00	6.32	11.73	8.48	7.78	5.68	8.00	1.93
Parietal inferior	0.68	0.00	1.69	1.30	1.25	1.74	1.11	0.61	9.33	10.27	11.17	12.18	12.57	9.71	10.87*	1.21
Occipital lateral	-1.88	-1.60	-1.09	1.18	0.60	-1.66	-0.74	1.19	4.35	3.26	0.00	1.17	1.20	1.68	1.94	1.44
Primary visual	-1.02	2.45	-1.74	2.58	-1.39	-1.40	-0.09	1.85	4.21	8.06	0.47	1.92	2.86	0.91	3.07	2.55
Temporal lateral	1.56	-2.44	0.64	1.45	-0.74	-2.04	-0.26	1.59	0.76	-0.61	1.87	0.71	0.00	1.32	0.68	0.81
Temporal mesial	0.99	-0.78	1.57	2.65	0.90	2.70	1.34	1.19	8.74	10.83	10.94	11.21	10.62	9.65	10.33*	0.86
Cerebellum	10.00	9.37	10.71	13.60	9.02	8.00	10.12*	1.77	10.00	9.37	10.71	13.60	9.02	8.00	10.12*	1.77
Pons	0.00	0.00	0.00	0.00	0.00	0.00	0.00	0.00	0.00	0.00	0.00	0.00	0.00	0.00	0.00	0.00

**ASD** = autism spectrum disorder; **Mean** = the average percentage change of brain glucose metabolism ratio among all ASD participants within the specified ROI; **SD** = the standard deviation of these values; $\:\mathbf{P}\mathbf{e}\mathbf{r}\mathbf{c}\mathbf{e}\mathbf{n}\mathbf{t}\mathbf{a}\mathbf{g}\mathbf{e}\:\mathbf{c}\mathbf{h}\mathbf{a}\mathbf{n}\mathbf{g}\mathbf{e}\:=\left(\frac{{\boldsymbol{B}\boldsymbol{G}\boldsymbol{M}}_{\boldsymbol{p}\boldsymbol{o}\boldsymbol{s}\boldsymbol{t}\boldsymbol{t}\boldsymbol{D}\boldsymbol{C}\boldsymbol{S}}-{\boldsymbol{B}\boldsymbol{G}\boldsymbol{M}}_{\boldsymbol{p}\boldsymbol{r}\boldsymbol{e}\boldsymbol{t}\boldsymbol{D}\boldsymbol{C}\boldsymbol{S}}}{{\boldsymbol{B}\boldsymbol{G}\boldsymbol{M}}_{\boldsymbol{p}\boldsymbol{o}\boldsymbol{s}\boldsymbol{t}\boldsymbol{t}\boldsymbol{D}\boldsymbol{C}\boldsymbol{S}}}\right)\times\:100$ ;*= clinically meaningful increase in glucose metabolism (> 10%)

**Table 3:** Comparison of regional brain glucose metabolism ratios between pre- and post-tDCS intervention and analysis of effect sizes (*N* = 6)

Region (ROI)	Pre-tDCS	Post-tDCS	% Change	Z-statistic	Unadjusted *p*-value*	FDR Adjusted *p*-value*	Effect Size (*r*)	Interpretation
	Mean ± SD	Mean ± SD						
Left Prefrontal Lateral	1.78 ± 0.14	1.99 ± 0.15	11.61%	2.23	0.026	0.027	0.91	Large
Left Parietal Inferior	1.69 ± 0.12	1.87 ± 0.14	10.87%	2.21	0.027	0.027	0.90	Large
Left Temporal Mesial	1.16 ± 0.08	1.28 ± 0.09	10.33%	2.21	0.027	0.027	0.90	Large
Cerebellum	1.27 ± 0.06	1.40 ± 0.08	10.12%	2.21	0.027	0.027	0.90	Large

Mean values represent the average brain glucose metabolism ratio in each ROI. P‑values were adjusted using the Benjamini–Hochberg correction to control the false discovery rate (FDR). Effect sizes of the correlation coefficient (Rosenthal’s r) of 0.1, 0.3, and 0.5 were interpreted as small, medium, and large, respectively. Unadjusted p‑values were tested using the Wilcoxon signed‑rank test

### Effect of tDCS on ATEC scores

Statistically significant reductions in ATEC scores were observed across all domains at 14 days post-intervention (Table 4). The mean total score decreased from a baseline of 55.83 to 36.83 (mean change − 19.00; *p* = 0.028), indicating a clinically meaningful reduction in observed scores.

**Table 4:** Changes in Autism Treatment Evaluation Checklist (ATEC) scores from baseline to Day 14 following tDCS intervention (*N* = 6)

ID	ATEC Language scores			ATEC Social scores			ATEC Sensory scores			ATEC Health scores			ATEC Total scores		
	Baseline	Day 14	Post-Pre	Baseline	Day 14	Post-Pre	Baseline	Day 14	Post-Pre	Baseline	Day 14	Post-Pre	Baseline	Day 14	Post-Pre
ASD01	8	5	-3	19	13	-6	15	13	-2	16	9	-7	58	40	-18
ASD02	13	10	-3	16	9	-7	18	15	-3	12	9	-3	59	43	-16
ASD03	15	14	-1	24	15	-9	20	16	-4	6	3	-3	65	48	-17
ASD05	8	6	-2	24	11	-13	13	8	-5	31	19	-12	76	44	-32
ASD06	0	0	0	9	2	-7	2	2	0	24	11	-13	35	15	-20
ASD07	5	3	-2	14	10	-4	16	13	-3	7	5	-2	42	31	-11
Mean	8.17	6.33	-1.83	17.67	10.00	-7.67	14.00	11.17	-2.83	16.00	9.33	-6.67	55.83	36.83	-19.00
SD	5.42	5.01	1.17	5.89	4.47	3.08	6.36	5.27	1.72	9.86	5.57	4.84	19.97	13.08	-6.90
p-value			0.041*			0.027*			0.042*			0.027*			0.028*

Outcomes are based on the Autism Treatment Evaluation Checklist (ATEC) assessed at baseline and at day 14 following transcranial direct current stimulation. The ATEC comprises four subscales: Language (14 items; maximum score, 28), Social (20 items; maximum score, 40), Sensory/Cognitive Awareness (18 items; maximum score, 36), and Health/Physical Behavior (25 items; maximum score, 75). The total ATEC score ranges from 0 to 179, with higher scores indicating greater symptom severity and lower scores reflecting clinical improvement. Severity categories are defined as follows: 0–30, very mild symptoms; 31–60, mild to moderate symptoms; 61–90, moderate to severe symptoms; and ≥ 91, very severe symptoms. An asterisk (*) indicates statistical significance by the Wilcoxon signed-rank test

Subscale analyses revealed reductions in: Social: Mean change − 7.67 (*p* = 0.027); Health/Physical: Mean change − 6.67 (*p* = 0.027); Sensory/Cognitive: Mean change − 2.83 (*p* = 0.042); Language: Mean change − 1.83 (*p* = 0.041).

### The association between changes in glucose metabolic ratios and clinical change

**Fig. 5:**
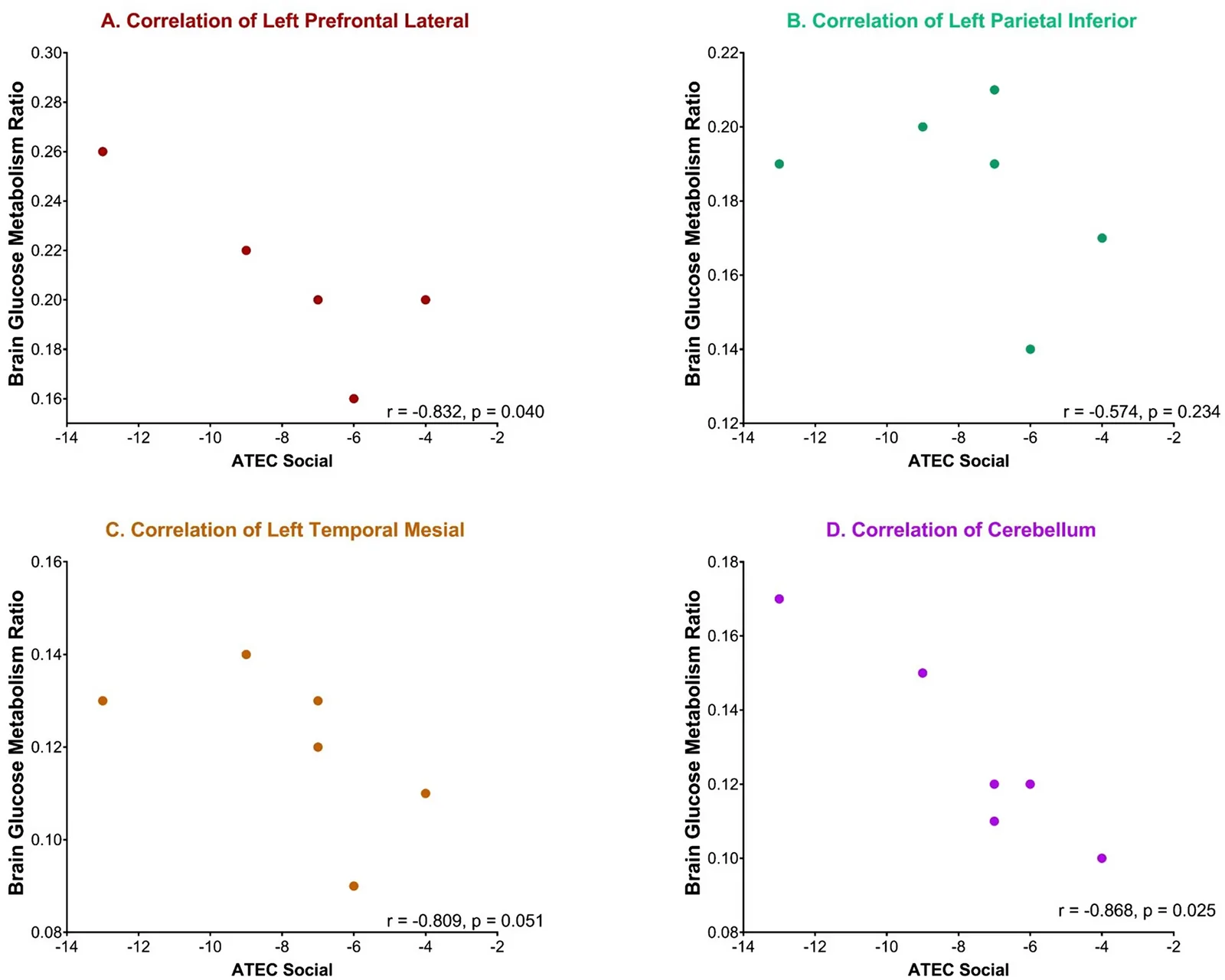
Scatter plots illustrate the relationship between brain glucose metabolism ratios and Autism Treatment Evaluation Checklist (ATEC) Social scores. (**A**) Left Prefrontal Lateral (**B**) Left Parietal Inferior (**C**) Left Temporal Mesial (**D**) Cerebellum. The coefficient r represents Spearman’s rho (p)

Guided by evidence of observed improvements following tDCS in social functioning [3–5], we examined the relationship between reductions in ATEC social scores and metabolic increases exceeding the predefined 10% threshold. Exploratory Spearman’s rank correlation analysis, conducted as hypothesis-generating analyses given the small sample size, revealed negative associations between ATEC social scores and metabolic increases in the left prefrontal lateral (*r* = − 0.832, *p* = 0.040) and cerebellum (*r* = − 0.868, *p* = 0.025) (Fig. 5). These correlations should be interpreted with caution, as individual participants may exert disproportionate influence on the correlation coefficient in a sample of *n* = 6. Scatter plots were examined and no single data point appeared to drive the association; however, this visual inspection cannot substitute for formal sensitivity analyses in larger samples. These findings are presented as preliminary observations requiring replication.

### Adverse events

No adverse events related to tDCS were reported in any of the participants.

## Discussion

The present pilot study elucidates the neurobiological underpinnings of the clinical improvements observed following anodal tDCS with an extracephalic reference in adolescents with ASD. Our results suggest that five sessions of 2-mA stimulation targeting the left DLPFC–right shoulder were associated with observed metabolic increases in cerebral glucose not only at the stimulation site but also in distal regions, including the inferior parietal and mesial temporal regions, and notably, the cerebellum. Crucially, these metabolic increases were showed preliminary negative associations with reductions in social deficits, suggesting that this montage may be associated with metabolic changes in nodes of a dysregulated “social brain” network.

### Effects of anodal tDCS on brain glucose metabolism: evidence from PET/CT imaging

The observed increase in cerebral glucose metabolism following anodal tDCS constitutes a preliminary finding of interest. To our knowledge, this is the first study to utilize PET/CT imaging to investigate these effects specifically within the autistic brain, underscoring the novelty of our approach. Although directly comparable data in autism is currently lacking, our results parallel tDCS-induced metabolic increases reported in other pathologies, including hemorrhagic stroke [29], multiple sclerosis [30], disorders of consciousness [31], cognitive impairment [20], Alzheimer’s disease [32], and neuropathic pain [33]. However, the magnitude of these metabolic changes varies across the literature, a discrepancy likely attributable to differences in electrode montages. Specifically, the present study employed an extracephalic reference (cathode on the right shoulder) with the anode over the left DLPFC, whereas prior studies [20, 29, 31–33] utilized intra-cephalic montages. Such variations in electrode placement critically alter the electric field distribution and current density, thereby modulating neuronal activity and subsequent regional glucose metabolism differently.

The metabolic increases observed are consistent with the use of an extracephalic montage (Left DLPFC–Right Shoulder) in delivering sufficient current density to the target cortex. Unlike conventional cephalic montages (e.g., F3-F4), where a significant portion of the current is shunted across the scalp, the extracephalic reference is thought to minimize superficial shunting and promote a deeper, more directional current flow through cortical and subcortical structures [15, 34].

### Mechanisms of tDCS-induced clinical change in ASD: a network perspective

Our study observed that anodal tDCS using the left DLPFC-right shoulder montage was associated with increases in brain glucose metabolism across the prefrontal lateral, parietal inferior, temporal mesial, and cerebellar regions. These findings suggest that the therapeutic effects of tDCS are not localized solely to the stimulation site but may potentially extend to critical nodes within distributed neuronal networks. Specifically, the specific montage used in this study could theoretically interact with the Frontoparietal Network (FPN), which is fundamental to attentional control and behavioral regulation [35].

The observed metabolic increase in the parietal inferior cortex—a core component of the mirror neuron system—offers a plausible hypothesis, rather than definitive evidence, regarding the improvements in social skills, given that this region underpins imitation and the understanding of others’ intentions [36]. Concurrently, metabolic changes in the temporal mesial region, encompassing the amygdala and hippocampus (key structures of the limbic system), might tentatively point toward a potential modulation of emotional processing and social memory circuits. Hypometabolism or hypoconnectivity in these specific areas has been frequently implicated in ASD pathophysiology [37].

Therefore, while our static, ROI-based data cannot directly verify dynamic connectivity, these localized findings are conceptually consistent with the hypothesis that anodal tDCS may exert a ‘top-down’ modulatory effect, potentially influencing these downstream nodes. However, further functional connectivity studies are warranted to confirm whether these regional metabolic alterations truly translate to a restoration of network-level synchronization, as previously suggested in reports of positive outcomes following tDCS over the temporoparietal junction [38].

### The role of the cerebellum: beyond motor control

A notable preliminary finding of this study is the observed metabolic increase in the cerebellum and its exploratory negative association (*r* = -0.868) with improved social interaction scores. While traditionally viewed as a motor control center, the cerebellum is increasingly recognized for its critical role in “social cognition,” specifically in processing social sequences and maintaining internal models of social interaction [39, 40]. The observed cerebellar activation is speculatively consistent with the modulation of cerebello-thalamo-cortical loops, which are known to be structurally and functionally disrupted in ASD [41]. By enhancing metabolic activity in the cerebellum, tDCS may potentially be associated with changes in the integrity of these long-range loops, thereby hypothetically relate to “social timing” and predictive processing deficits characteristic of ASD. To our knowledge, direct cerebellar stimulation remains an underexplored avenue in ASD research. Our findings suggest that the cerebellum represents a promising novel target for future neuromodulatory interventions aiming to reduce ASD severity.

### Inter-individual variability in response

It is noteworthy that while exceeding the predefined 10% threshold increases in the lateral prefrontal cortex were universally observed in all participants (*n* = 6/6), similar increases in distal regions—specifically the inferior parietal, mesial temporal, and cerebellar areas—were detected in 5 out of 6 participants. This variability likely stems from inter-individual anatomical differences, such as skull thickness and gyrification patterns, which are known to influence the spatial distribution and intensity of the induced electric field across different brain regions [42, 43].

### Limitations

While this study provides novel insights into the neurometabolic correlates of tDCS in adolescents with ASD, several methodological caveats must be acknowledged.

First, the primary constraint of this investigation is its open-label design, coupled with a small sample size (*n* = 6) and the absence of a parallel, randomized sham-controlled group. Given that the clinical behavioral outcomes were evaluated via the ATEC-a subjective, caregiver-reported instrument-the findings are inherently vulnerable to parental expectancy bias, placebo effects, and potential confounding from natural developmental progression or regression-to-the-mean. Consequently, these behavioral trends cannot be interpreted as definitive evidence of therapeutic efficacy. To mitigate these biases, strict procedural controls were implemented, including the utilization of a fully automated quantitative PET analysis pipeline executed by a nuclear medicine physician blinded to individual clinical profiles, alongside standardized assessment delivery. Nonetheless, future large-scale, double-blind, sham-controlled trials utilizing objective, clinician-rated instruments (such as the Autism Diagnostic Observation Schedule, Second Edition [ADOS-2]) are fundamentally required to isolate the true therapeutic potency of this stimulation protocol.

Second, a distinct temporal decoupling exists between our two primary outcome measures: metabolic neuroimaging was captured 1-day post-intervention, whereas behavioral phenotypic changes were assessed at Day 14. Although this timeline was strategically selected based on clinical precedents-aiming to capture acute, direct neurometabolic modulation prior to washout while allowing sufficient time for cumulative behavioral improvements to stabilize-this cross-sectional asymmetry precludes the establishment of a direct, concurrent causal link. Future longitudinal studies incorporating multi-time-point or mid-intervention neuroimaging assessments are warranted to delineate the sequential trajectory from immediate metabolic reconfiguration to delayed behavioral consolidation.

Third, the operational threshold of > 10% metabolic change for ROI selection was extrapolated from established test–retest reliability data in oncological literature rather than being a priori validated within an ASD neuromodulation paradigm. While this stringent threshold was deliberately adopted as a conservative filter to minimize Type I errors and filter out baseline physiological variance or technical scanner noise within a restricted sample, its generalizability to neurodevelopmental metabolic kinetics remains preliminary. These findings should thus be interpreted with caution until larger, disorder-specific validation cohorts establish standardized neuromodulatory metabolic thresholds.

Finally, the FDR correction was confined strictly to the subset of ROIs that breached the initial > 10% magnitude threshold, rather than being applied across all 26 predefined ROIs simultaneously. Although this two-stage analytical pipeline was implemented to balance statistical stringency with the limited statistical power inherent in a pilot cohort, this conditional approach introduces a risk of selection bias (circular analysis or double-dipping). As a result, the reported p-values should be considered exploratory and hypothesis-generating. Confirmatory trials with larger, adequately powered cohorts should apply multiple-comparison corrections across the entire ROI set a priori to ensure ultimate statistical rigor.

## Conclusion

This open-label pilot study provides preliminary metabolic imaging evidence that five sessions of anodal tDCS delivered via a left DLPFC–right shoulder montage are associated with regional increases in cerebral glucose metabolism—most notably in the prefrontal lateral and cerebellar regions—that correlate with reductions in caregiver-rated social scores in adolescents with ASD. These findings are exploratory and hypothesis-generating. Given the small sample, absence of a sham control group, open-label design, and analytic limitations described above, no causal inferences regarding therapeutic efficacy can be drawn. The primary value of this work lies in identifying candidate neural substrates for this extracephalic montage and in providing effect-size estimates to inform sample-size calculations for future studies. We recommend that these observations be evaluated in larger, randomized, sham-controlled trials incorporating blinded clinical assessors, multiple PET time points, and voxel-level statistical mapping.

## Supplementary Information

Below is the link to the electronic supplementary material.

Supplementary Material 1

## Data Availability

All data generated or analysed during this study are included in this published article and its supplementary information files.

## Abbreviations

ASD: Autism Spectrum Disorder
DLPFC: Dorsolateral prefrontal cortex
tDCS: Transcranial direct current stimulation
PET/CT: Positron emission tomography/computed tomography
MRI: Magnetic resonance imaging
