# Supplementary material for: Increased brain glucose metabolism after anodal left DLPFC–right shoulder tDCS in ASD: an open-label pilot F-18 FDG PET/CT study
Source: BMC Psychiatry. 2026 Jul 20;26:793. doi: 10.1186/s12888-026-08418-x (PMC13670782; doi:10.1186/s12888-026-08418-x)
Supplement: Supplementary file 1 [file 12888_2026_8418_MOESM1_ESM.docx]

| **Raw data: Quantitative assessment in brain glucose metabolism ratios across regions of interest in the ASD group (N = 6) derived from Q.Brain software.** | | | | | | | | | | | | | | | | | |
| --- | --- | --- | --- | --- | --- | --- | --- | --- | --- | --- | --- | --- | --- | --- | --- | --- | --- |
| **Region of interest (ROI)** | | **Right Hemisphere** | | | | | | | | **Left Hemisphere** | | | | | | | |
|  |  | **ASD01** | **ASD02** | **ASD03** | **ASD05** | **ASD06** | **ASD07** | **Mean** | **SD** | **ASD01** | **ASD02** | **ASD03** | **ASD05** | **ASD06** | **ASD07** | **Mean** | **SD** |
| **Prefrontal Lateral** | Pre | 1.50 | 1.88 | 1.96 | 1.69 | 1.64 | 1.80 | 1.75 | 0.15 | 1.54 | 1.91 | 1.96 | 1.72 | 1.73 | 1.84 | 1.78 | 0.14 |
|  | Post | 1.55 | 1.98 | 2.01 | 1.75 | 1.67 | 1.88 | 1.81 | 0.17 | 1.70 | 2.11 | 2.18 | 1.98 | 1.93 | 2.04 | 1.99 | 0.15 |
| **Prefrontal Medial** | Pre | 1.43 | 1.87 | 1.81 | 1.67 | 1.57 | 1.60 | 1.66 | 0.15 | 1.44 | 1.79 | 1.82 | 1.56 | 1.63 | 1.74 | 1.66 | 0.13 |
|  | Post | 1.49 | 1.92 | 1.98 | 1.68 | 1.58 | 1.67 | 1.72 | 0.18 | 1.55 | 1.92 | 1.99 | 1.64 | 1.71 | 1.77 | 1.76 | 0.15 |
| **Sensorimotor** | Pre | 1.76 | 2.03 | 1.88 | 1.78 | 1.96 | 1.96 | 1.90 | 0.10 | 1.73 | 2.00 | 1.89 | 1.79 | 2.01 | 1.97 | 1.90 | 0.11 |
|  | Post | 1.74 | 1.99 | 1.91 | 1.72 | 1.94 | 2.03 | 1.89 | 0.12 | 1.77 | 2.04 | 1.97 | 1.85 | 2.08 | 2.06 | 1.96 | 0.11 |
| **Anterior cingulate** | Pre | 1.31 | 1.60 | 1.71 | 1.52 | 1.48 | 1.71 | 1.56 | 0.14 | 1.30 | 1.64 | 1.74 | 1.54 | 1.45 | 1.71 | 1.56 | 0.15 |
|  | Post | 1.32 | 1.58 | 1.68 | 1.5 | 1.49 | 1.65 | 1.54 | 0.12 | 1.31 | 1.65 | 1.75 | 1.60 | 1.58 | 1.72 | 1.60 | 0.14 |
| **Posterior cingulate** | Pre | 1.69 | 2.08 | 1.88 | 1.84 | 1.85 | 1.92 | 1.88 | 0.12 | 1.70 | 2.00 | 1.89 | 1.80 | 1.91 | 1.95 | 1.88 | 0.10 |
|  | Post | 1.68 | 2.1 | 1.91 | 1.86 | 1.84 | 1.93 | 1.89 | 0.12 | 1.71 | 2.06 | 1.92 | 1.86 | 1.92 | 1.96 | 1.91 | 0.11 |
| **Precuneus** | Pre | 1.48 | 1.74 | 1.84 | 1.58 | 1.60 | 1.71 | 1.66 | 0.12 | 1.52 | 1.76 | 1.84 | 1.63 | 1.61 | 1.81 | 1.70 | 0.12 |
|  | Post | 1.44 | 1.73 | 1.82 | 1.61 | 1.61 | 1.68 | 1.65 | 0.12 | 1.54 | 1.79 | 1.86 | 1.72 | 1.65 | 1.84 | 1.73 | 0.11 |
| **Parietal superior** | Pre | 1.51 | 1.84 | 1.61 | 1.58 | 1.71 | 1.73 | 1.66 | 0.11 | 1.50 | 1.90 | 1.62 | 1.65 | 1.67 | 1.76 | 1.68 | 0.12 |
|  | Post | 1.62 | 1.87 | 1.68 | 1.6 | 1.77 | 1.74 | 1.71 | 0.09 | 1.62 | 2.02 | 1.81 | 1.79 | 1.80 | 1.86 | 1.82 | 0.12 |
| **Parietal inferior** | Pre | 1.47 | 1.83 | 1.77 | 1.54 | 1.60 | 1.72 | 1.66 | 0.13 | 1.50 | 1.85 | 1.79 | 1.56 | 1.67 | 1.75 | 1.69 | 0.12 |
|  | Post | 1.48 | 1.83 | 1.8 | 1.56 | 1.62 | 1.75 | 1.67 | 0.13 | 1.64 | 2.04 | 1.99 | 1.75 | 1.88 | 1.92 | 1.87 | 0.14 |
| **Occipital lateral** | Pre | 1.60 | 1.87 | 1.84 | 1.69 | 1.68 | 1.81 | 1.75 | 0.10 | 1.61 | 1.84 | 1.83 | 1.71 | 1.67 | 1.79 | 1.74 | 0.08 |
|  | Post | 1.57 | 1.84 | 1.82 | 1.71 | 1.69 | 1.78 | 1.74 | 0.09 | 1.68 | 1.90 | 1.83 | 1.73 | 1.69 | 1.82 | 1.78 | 0.08 |
| **Primary visual** | Pre | 1.96 | 2.04 | 2.30 | 1.94 | 2.16 | 2.15 | 2.09 | 0.13 | 1.90 | 1.86 | 2.15 | 2.08 | 2.10 | 2.20 | 2.05 | 0.13 |
|  | Post | 1.94 | 2.09 | 2.26 | 1.99 | 2.13 | 2.12 | 2.09 | 0.10 | 1.98 | 2.01 | 2.16 | 2.12 | 2.16 | 2.22 | 2.11 | 0.09 |
| **Temporal lateral** | Pre | 1.28 | 1.64 | 1.57 | 1.38 | 1.35 | 1.47 | 1.45 | 0.13 | 1.32 | 1.65 | 1.60 | 1.40 | 1.42 | 1.52 | 1.49 | 0.12 |
|  | Post | 1.30 | 1.60 | 1.58 | 1.40 | 1.34 | 1.44 | 1.44 | 0.11 | 1.33 | 1.64 | 1.63 | 1.41 | 1.42 | 1.54 | 1.50 | 0.12 |
| **Temporal mesial** | Pre | 1.01 | 1.28 | 1.27 | 1.13 | 1.11 | 1.11 | 1.15 | 0.10 | 1.03 | 1.20 | 1.28 | 1.16 | 1.13 | 1.14 | 1.16 | 0.08 |
|  | Post | 1.02 | 1.27 | 1.29 | 1.16 | 1.12 | 1.14 | 1.17 | 0.09 | 1.12 | 1.33 | 1.42 | 1.29 | 1.25 | 1.25 | 1.28 | 0.09 |
| **Cerebellum** | Pre | 1.20 | 1.28 | 1.40 | 1.25 | 1.22 | 1.25 | 1.27 | 0.06 | 1.20 | 1.28 | 1.40 | 1.25 | 1.22 | 1.25 | 1.27 | 0.06 |
|  | Post | 1.32 | 1.40 | 1.55 | 1.42 | 1.33 | 1.35 | 1.40 | 0.08 | 1.32 | 1.40 | 1.55 | 1.42 | 1.33 | 1.35 | 1.40 | 0.08 |
| **Pons** | Pre | 1.00 | 1.00 | 1.00 | 1.00 | 1.00 | 1.00 | 1.00 | 0.00 | 1.00 | 1.00 | 1.00 | 1.00 | 1.00 | 1.00 | 1.00 | 0.00 |
|  | Post | 1.00 | 1.00 | 1.00 | 1.00 | 1.00 | 1.00 | 1.00 | 0.00 | 1.00 | 1.00 | 1.00 | 1.00 | 1.00 | 1.00 | 1.00 | 0.00 |
